# Rational synthesis of atomically thin quantum structures in nanowires based on nucleation processes

**DOI:** 10.1038/s41598-020-67625-y

**Published:** 2020-07-01

**Authors:** Katsuhiro Tomioka, Junichi Motohisa, Takashi Fukui

**Affiliations:** 1 Graduate School of Information Science and Technologyand Research Center for Integrated Quantum Electronics (RCIQE), Hokkaido University, Kita 13 Nishi 8, Sapporo, 060-8628 Japan; 2 Japan Science and Technology Agency (JST), PRESTO, Kawaguchi, Saitama 332-0012 Japan

**Keywords:** Nanoscience and technology, Nanoscale materials

## Abstract

Excitonic properties in quantum dot (QD) structure are essential properties for applications in quantum computing, cryptography, and photonics. Top-down fabrication and bottom-up growth by self-assembling for forming the QDs have shown their usefulness. These methods, however, still inherent issues in precision integrating the regimes with high reproducibility and positioning to realize the applications with on-demand quantum properties on Si platforms. Here, we report on a rational synthesis of embedding atomically thin InAs in nanowire materials on Si by selective-area regrowth. An extremely slow growth rate specified for the synthesis demonstrated to form smallest quantum structures reaching nuclear size, and provided good controllability for the excitonic states on Si platforms. The system exhibited sharp photoluminescence spectra originating from exciton and bi-exciton suggesting the carriers were confined inside the nuclei. The selective-area regrowth would open new approach to integrate the exciton states with Si platforms as building-blocks for versatile quantum systems.

## Introduction

Semiconductor quantum dots (QDs) have attracted much attention as a light source using single and entangled photons in quantum cryptography^1,2^. The typical method of growing QDs is the self-assembled method using Stranski–Krastnov (S–K) mode due to lattice mismatch^3^. This growth method, however, has several challenges in controlling size, density, and positions and the S–K mode has been restricted in typical heterostructures such as In(Ga)As/GaAs systems. This limits the possibility of using optically active QDs on Si platforms for future Si-based optical circuits with versatile quantum system.

Recent progresses in epitaxial methods, such as vapor–liquid–solid method and selective-area epitaxy, have resulted in the growth of axial heterostructures in nanowires (NWs) regardless of lattice mismatch, i.e., various materials, such as quantum disks and QDs, could be embedded inside host NWs^4–8^. NW axial heterostructures have great potential for future quantum optical applications because flexible band-gap engineering is possible, and the diameter, height, and density of QDs can be controlled by modifying the host NW’s geometry. NW axial heterostructures have been fabricated in axial InP/InAs^9^, GaAs/GaP^10^, and GaAs/GaAsSb NWs^11^. These quantum structures embedded in NWs allowed single-electron transport^9^, resonant tunneling regime^9^, strong anti-bunching^12^, and exciton spin memory^13^. Moreover, tuning of optically active QDs’ charge state using gate voltage has been achieved using these NWs^14^. The GaAs/InAs system as quantum light source, which operated at near-infrared wavelength region, are quite feasible for quantum system on-chip integration with Si based avalanche photo diodes inside Si microchips. These state-of-the-art phenomena induced by the quantum structures with GaAs/InAs axial heterostructures should be integrated on Si platforms as a future element in Si-CMOS compatible optical circuits using quantum cryptosystems.

In this regard, integration of QDs with Si and light-emitting diodes on GaAs/Si wafer-bonded substrates have been reported^15,16^. Embedding quantum structures into the host NW materials regardless of abnormal deposition on NW sidewalls is required for precise controlling and tuning of the quantum states on Si platforms. And the NW with nanoscale footprints would be expected to have strong confinement either or both in the axial and radial direction, which allows robustness and reproducibility of the excitonic properties. Thus, synthesis of an atomically thin quantum structure is essential for strong confinement. In addition, precise positioning of the structures is also important for fabricating on-demand light-source devices. Recent advances in selective-area epitaxy have enabled integration of vertical GaAs NWs and light sources on Si substrates^17,18^, and templated assisted selective epitaxy (TASE)^19,20^, which uses Si microstructures as templates, enables the integration of III-Vs nanomaterials on Si nanostructures such as electronic^21^ and optical^22^ devices on Si platforms. By using GaAs NWs grown using selective-area epitaxy as templates for regrowth, atomically thin InAs layers can be embed inside these thin NWs. Therefore, we characterized vertical stacked GaAs/InAs/GaAs heterostructures in position-controlled GaAs NWs on Si by the selective-area regrowth.

## Results

### Selective-area regrowth of atomically-thin InAs embedded GaAs NWs on Si substrates

We demonstrated selective-area regrowth to embed four InAs layers inside vertical GaAs NWs on Si. This method prevents unintentional growth on the sidewalls of host NWs by covering the sidewalls with amorphous (SiO_2_) films. We discuss the growth morphology of atomically-thin InAs layers by the selective-area regrowth. The grown InAs layers exhibited sharp photoluminescence (PL), indicating the formation of InAs quantum structures (disks or dots). And excitation power dependence on the PL intensity revealed that the InAs atomically thin layer via nucleation of two-dimensional (2D) InAs islands resident exciton and bi-exciton.

**Figure 1:**
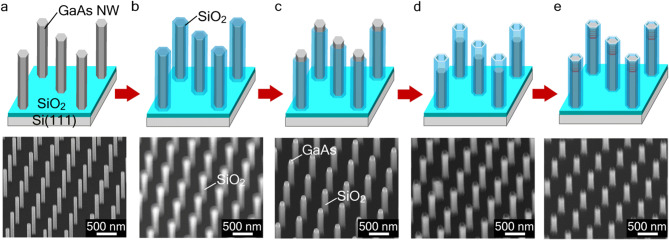
Selective-area regrowth. (**a**) Selective-area growth (SAG) of vertical GaAs NWs on Si. (**b**) Deposition of SiO_2_ by RF sputtering. (**c**) RIE of top portion of SiO_2_ film. (**d**) Selective wet etching to form SiO_2_ tube. (**e**) Regrowth of GaAs/InAs/GaAs heterostructures by pulse growth modes.

Vertical GaAs NWs were grown on Si(111) by using selective-area epitaxy by changing the initial Si surface into a (111)B-polar surface^17^. The vertical GaAs NWs array was successfully integrated on the Si(111) surface by the selective-area epitaxy (Fig. 1a). The design of the GaAs/InAs/GaAs axial heterostructures consisted of four InAs layers, the growth cycle of each InAs was 2, 6, 10, and 20 cycles with 10-nm-thick GaAs barriers. The processes of selective-area regrowth were as follows. First, 100-nm-thick SiO_2_ was deposited on the vertical GaAs NWs, as shown in Fig. 1b. In the sputtering procedure, the sidewalls of the GaAs NWs were covered with 50-nm-thick SiO_2_. Next, reactive-ion-etching (RIE) was carried out to etch the top portion of the SiO_2_-covered NWs (Fig. 1c). Then, the top part of the NWs were selectively etched using NH_4_OH:H_2_O_2_:H_2_O = 5:1:1 solution. This etching process was used to form a SiO_2_ cylinder on the GaAs NWs, as illustrated in Fig. 1d. The etching solution selectively formed GaAs(111)A surface inside the SiO_2_ cylinder. Then, GaAs buffer layer was grown under the same growth condition of GaAs NW in order to recover the GaAs(111)B top surface. Finally, GaAs/InAs/GaAs vertical stacked layers were grown by pulsed growth with different InAs cycles, as shown in Fig. 1e. The purpose of the pulsed growth for the GaAs/InAs/GaAs layers was to elongate the surface diffusion length of the Ga and In atoms inside the SiO_2_ nanotube and avoid abnormal deposition outside the sidewalls of the SiO_2_^23^.

### Photoluminescence of the atomically-thin InAs structures

**Figure 2:**
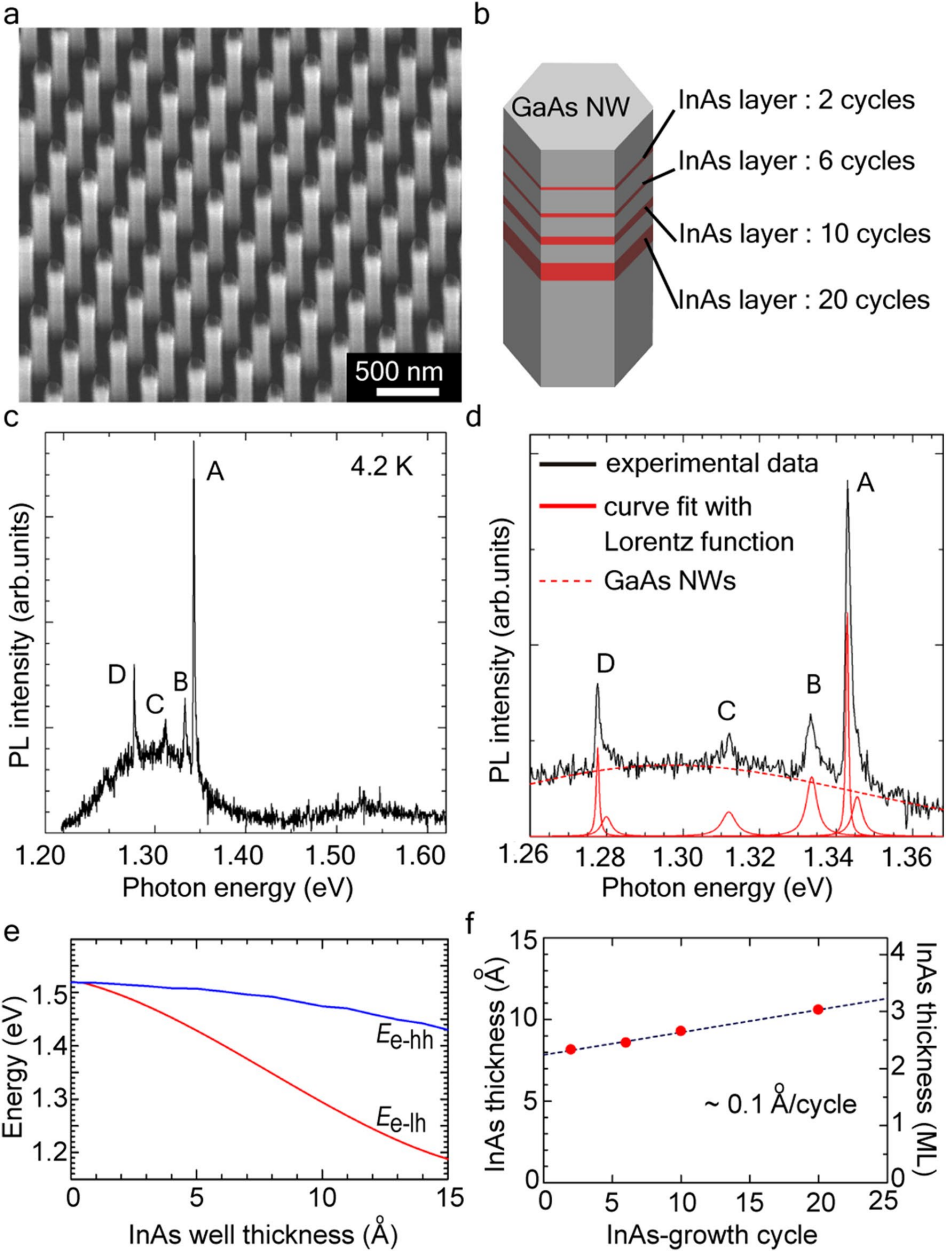
Atomically thin InAs layers-embedded GaAs NWs on Si. (**a**) 45°-tilted SEM image showing the InAs-embedded GaAs NW array on Si. (**b**) Illustration of GaAs/InAs/GaAs multilayers grown using the selective-area regrowth. (**c**) μ-PL spectra for GaAs/InAs/GaAs NWs on Si measured at 4.2 K. (**d**) Peak deconvolution of the PL spectra using Lorentzian function. (**e**) Plot of transition energy for heavy-hole and light-holes as a function of InAs thickness for GaAs/InAs/GaAs strained QW on GaAs. (**f**) Plot of InAs thickness with variation in the pulse cycles.

Figures 2a showed the growth results of the InAs atomically-thin layer embedded GaAs NWs on Si substrate. We designed InAs layers with four different numbers of cycle, as illustrated in Fig. 2b. A uniformly SiO_2_-coated GaAs NW consisting of the four InAs layers, illustrated in Fig. 2b, was integrated on the Si substrates. The NW diameter was 100 nm, height was approximately 1 μm, and diameter fluctuation was ± 4 nm. There were no abnormal depositions on the SiO_2_ sidewalls, which indicated the source materials react on only the (111)B surface surrounded by the SiO_2_ tube. The micro (μ)-PL spectra under low excitation power in Fig. 2c showed sharp optical emissions around 1.30 eV at the excitation power of 0.92 kW/cm^2^. The number of sharp PL peaks corresponded to the number of InAs layers. The other PL peaks with broad full width at half maximum (FWHM) at around 1.30 eV were assumed to be deep level emission from the GaAs NWs.

The positions of the four sharp PL peaks were 1.342 (peak A), 1.334 (peak B), 1.312 (peak C), and 1.277 eV (peak D). These PL peak positions were almost constant at arbitral positions in the GaAs/InAs/GaAs NW array (see in Supplementary Fig. S1). This means the size fluctuation of the InAs layer is significantly small in the regrowth process. We analyzed the PL spectra by using Lorentzian functions for the sharp PL and a Gaussian function for the broaden PL. The curve-fitting with Lorentzian function estimated FWHM of 630 μeV for peak A, 1.2 meV for peak B, 1.7 meV for peak C, and 980 μeV for peak D shown in Fig. 2d. These PL properties exhibited similar luminescence behavior to the other InAs QDs which was formed by S–K growth and selective-aera growth (see in Supplementary Table S1).

Figure 2e shows the calculated transition energy level in a strained GaAs/InAs single QW. We ascribed the InAs QW on GaAs(111)^24^ and excluded the quantum confinement effect in the radial direction. Assuming the sharp PL peaks in Fig. 2c originated from the InAs layers, the InAs thickness was linearly changed with increasing the InAs cycle (Fig. 2f). The growth rate of the InAs layers was estimated to be 0.1 Å/s, which was extremely slower than 1 monolayer [ML, 3.498 Å for InAs(111)]. This indicates that the InAs ML-growth proceed with very slow growth rate involving a step flow growth in the SiO_2_ tubes.

## Discussions

### Nucleation growth process in selective-area regrowth of the atomically thin InAs layers

**Figure 3:**
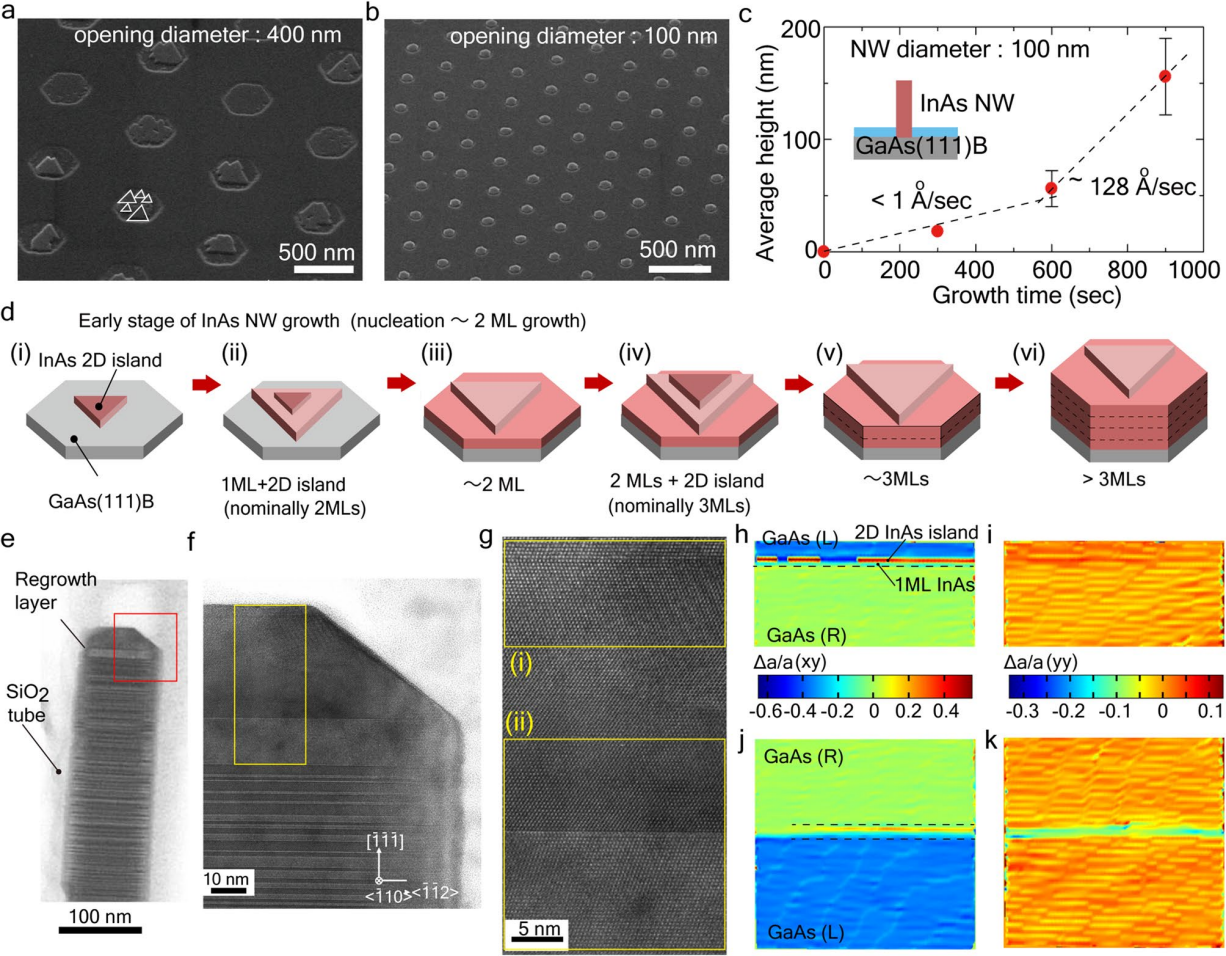
Growth morphologies and footprint of atomically thin InAs layers in selective-area regrowth. SEM images showing SAG of InAs on GaAs (111)B substrate with (**a**) 400-nm and (**b**) 100-nm opening diameters. (**c**) Average height of InAs NWs grown by SAG. Opening diameter was 100 nm. Dashed lines are guide lines for each growth rate. (**d**) Illustrations of early and middle stages of atomically thin InAs growth in selective-area regrowth: (i) nucleation of InAs 2D island, (ii) nucleation of 2D InAs island on 1 ML-thick InAs large island, (iii) I ML growth and 2D InAs island step-flow growth, (iv) nucleation on 2 ML-thick InAs layer, (v) formation of 3 ML-thick InAs layer (complete 2 ML with incomplete InAs ML), (vi) Growth of InAs layers. (**e**) TEM image of atomically thin InAs layer embedded GaAs NW by selective-area regrowth. (**f**) Magnified TEM image of red square in panel (**e**). (**g**) High-resolution TEM image adjacent to GaAs/InAs layers shown in yellow square in panel (**f**,**h**). ε_xy_ strain mapping estimated from filtered image of yellow square (i) in panel (**g**). GaAs(L) and GaAs(R) stands for atomic layer zinc-blende (ZB) stacking along right and left side. The lattice constant for the GaAs(R) was standards for the strain estimation. 1 ML InAs imcomplete ML and 2D InAs islands were observed. (**i**) ε_yy_ strain mapping estimated from filtered image of yellow square (i) in panel (**g**). (**j**,**k**) ε_xy_ and ε_yy_ strain mapping estimated from filtered image of yellow square (ii) in panel (**g**). 2ML-thick InAs and 1ML-thick large 2D InAs island was observed in each panel.

We investigated the early stage of InAs SAG on GaAs(111)B surface to reveal the extremely slow rate in the InAs regrowth process. Figure 3a,b show the SAG of InAs on GaAs(111)B surface, the growth time of which was 5 min. Growth condition for the InAs was almost same as InAs layer (explained in method), but the gas supply was continuous flow condition without pulsed growth. Figure 3a illustrates the coalescence of triangular-shaped InAs islands on large openings with a opening diameter of 400 nm. On the other hand, hexagonal-shaped structures were formed on openings with a diameter of 100 nm (Fig. 3b). In case of conventional SAG, the nominal growth rate was approximately below 1 Å/s for InAs (and 10 nm/min for GaAs) which was estimated from that on SAG of InAs (GaAs) NWs on GaAs(111)B substrates. Although the growth in the InAs/GaAs lattice mismatched system proceeded with the S–K mode involving a wetting layer, the InAs on the GaAs(111)A surface exhibited formation of 2D stacking fault tetrahedron^25^. The InAs nucleation process of SAG on GaAs(111)B thought to follow the formation of the 2D islands.

Similar growth morphology was observed in the other heteroepitaxial system in such GaAs/Si (lattice mismatch is about 4.1%)^17^. Another investigation of STM reported that the formation of 2D InAs islands^26^. The 2D InAs islands have flat (111)B surfaces, and the initial stage of the InAs NW growth on GaAs(111)B was based on the coalescence of these InAs 2D islands because multinuclear growth and next stacking proceed before the InAs monolayer (ML) is fulfilled the whole surface in the openings. Note that a part of the initial GaAs(111)B surface thought to have As-trimer^27^ resulting in a slow nucleation rate due to the surface reconstruction. And the InAs crystallization gradually starts from the steps of the InAs 2D islands. Figure 3c shows the average height of InAs NWs as a function of growth time, indicating the height was sub-linearly increased with growth time due to the above growth process. The dashed lines are guide lines of 1 Å/s and 128 Å/s, respectively. In the early growth stage, the growth rate was much smaller than the 1 Å/s, which coincides to the growth rate estimated in Fig. 3b.

Figure 3d illustrates growth evolution of the atomically thin InAs layer. With increasing the number of pulse cycle for InAs regrowth, the first nuclei (2D InAs island) eventually form a complete ML through the step-flow growth. The surface diffusion of In atoms was dominant supply process as compared to Knudsen diffusion inside the SiO_2_ in TASE method^28^. Same condition occurred in the regrowth. However, the multinuclear growth as similar to the high supersaturation condition would repeat until the 2D InAs island evolves into complete monolayer due to extremely small step-flow growth rate. In this case, the step-flow growth-rate for the one ML became slow because the InAs islands and each step compete for diffused In atoms.

The InAs thickness estimated from the PL peaks and their extreme slow growth rate evidenced this growth process. Approximately 2 ML-thick InAs layer estimated from the PL peak A indicates that the InAs layer illustrated in Fig. 3d(ii) was formed and electron–hole pair was confined inside the 2D island due to overlapping each wavefunction. When the InAs pulse cycle was 10, 2ML-thick with 2D InAs islands as illustrated in Fig. 3d(v) were formed through the growth process and the PL emission corresponds to three ML-thick InAs layer. Therefore, the selective-area regrowth is able to form atomically thin InAs layer by changing the pulse cycles, and enables the formation of quantum structures using 2D InAs nucleus with arbitral size and position, which is difficult for the conventional S–K growth mode.

The GaAs NW composed of three different InAs layers were fabricated by this method in order to clearly characterize the atomically thin layer by TEM. The regrowth for the InAs layers were 9, 18, and 30 cycles. Sharp PL peaks reflecting the three InAs layers were also observed (see in Supplementary Fig. S4). Figure 3e–k show representative TEM image and strain mappings. The crystal phase of the host GaAs NW was a zinc-blende (ZB) structure with twining rotation. The GaAs layer had a few twining rotations in the regrowth layer, and single crystal ZB phase was elongated, which indicates extremely slow growth rate with a low supersaturation^29^ and avoidance of a step edge roughening^30^ affected to the GaAs regrowth layers.

The strain mappings estimated from the peak-pair-finding method for the adjacent InAs layer [yellow squares (i) and (ii) in Fig. 3g] are shown in Fig. 3h–k. First, 1 ML-thick InAs and 2D InAs islands (1 ML-thick), which was assumed in Fig. 3d(iii), were observed in the vicinity of the top surface. The 2D InAs island showed around 3–10-nm-width lamellar strain, which are never explained only by the rotation twin. The width of the lamellar strain field was covered with a section of the whole surface. This abnormal strain field thus suggested the formation of ML-thick 2D InAs islands, which was speculated in Fig. 3d. In case of InAs layer grown with 18 cycles [the yellow square (ii) in Fig. 3g], the similar lamellar strain estimated from the 3ML-thick InAs was observed. These images indicated that the second 2D InAs island was elongated on the 2ML-thick InAs layer via step-flow growth, which was ascribed in Fig. 3d(v).

### Optical transition process for the atomically thin InAs

**Figure 4:**
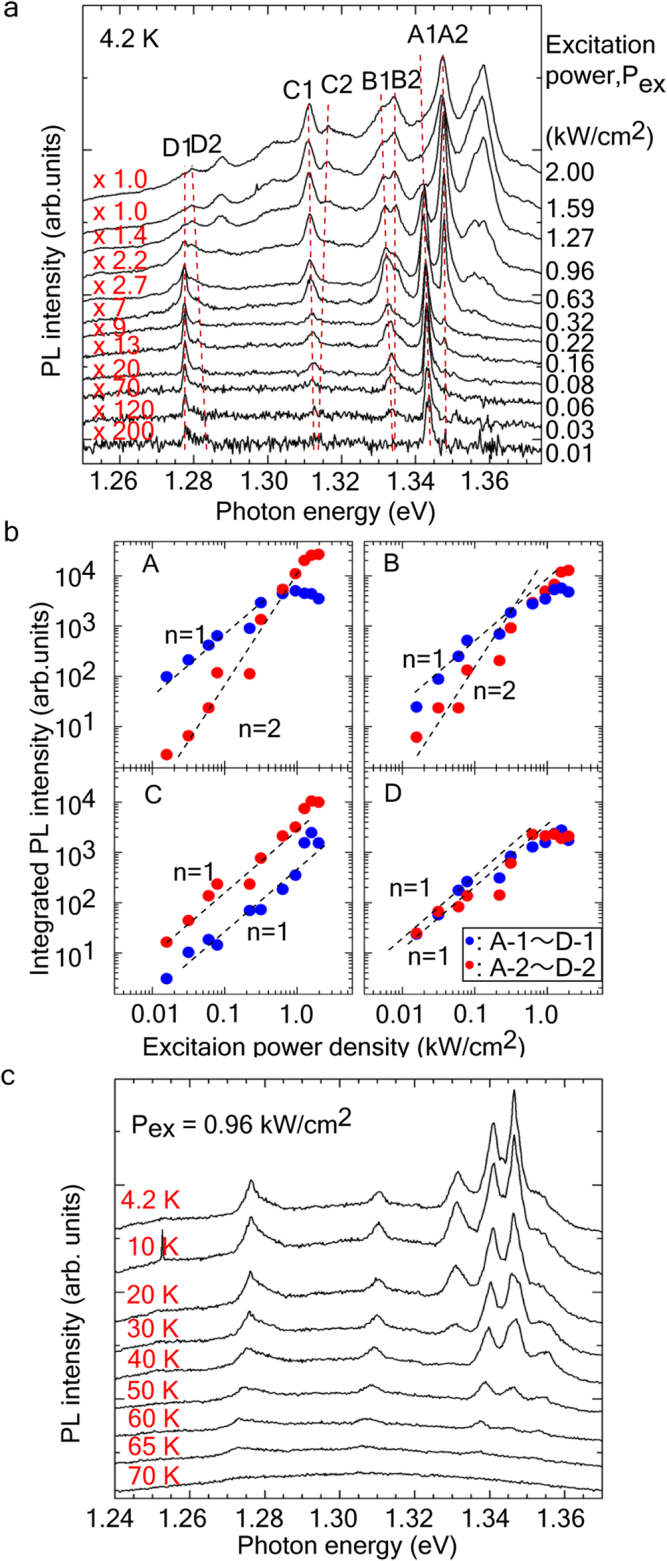
Optical properties for the atomically thin InAs layers in GaAs NWs. (**a**) Excitation-power dependence of μ-PL spectra of GaAs/InAs/GaAs NWs on Si. (**b**) Integrated PL intensity for bands A, B, C, and D as a function of excitation-power density. (**c**) Temperature dependence of μ-PL spectra. Excitation power density was 0.96 kW/cm^2^.

Figure 4a shows the excitation power dependence of each PL band. These PL bands showed double-splitting with increasing excitation power density. This splitting relates to exciton/biexciton or ground-state/higher state levels in InAs layers. Figure 4b shows the integral PL intensity for each PL band as a function of excitation power density. The blue circles are the lower positions of PL spectra (A1 ~ D1). The red circles are the higher peaks (A2 ~ D2). In the PL spectra (A and B), the PL intensities in A1 and B1 monotonically increased with increasing excitation power density. This excitation-power dependence indicates the optical transition originating from free exciton emission. The PL intensities for A2 and B2, however, quadratically increased with increasing the excitation power density, meaning biexciton emission was involved. These behaviors of the exciton and biexciton emissions were similarly observed in other NWs such as InAsP quantum dots embedded in InP NWs^31^ and InAs QDs grown on GaAs NWs^32^.

The energy level of biexciton is typically lower than that of exciton because of the positive binding energy for biexciton resulting from attractive Coulomb interaction. However, the biexciton in the regrown InAs layer had negative binding energy, exhibiting a higher energy state than that of exciton. This means that the Coulomb interaction of the two localized excitons is repulsive due to the narrow confinement space. The negative binding energy was 10 meV in InAs QDs^33^ and the energy depends on the shapes of the QDs^34^. The energy difference between the exciton and biexciton for bands A and B at an excitation power of 0.96 kW/cm^2^ was − 6 and − 3 meV, respectively. The emission of A and B had strong confinement as QDs and thought to be originated from the InAs layer grown with 2 and 6 cycles.

The PL properties in band A indicated the formation of an InAs layer grown with 2 cycles involving 2D InAs island on InAs 1 ML. In this case, triangle-shaped 2D InAs island emitted sharp emission because excited carriers in the InAs layer were collected inside the smaller 2D InAs island due to overlapping of the carrier wavefunctions. Hence, biexciton emission was observed in case of the InAs layer. Additional peaks appeared at higher energy compared with band A by further increasing excitation power. These additional PL peaks were thought to be multiple exciton emission due to the lower dimensionality. For the PL peaks at bands C and D in Fig. 4a, the entire PL intensity monotonically increased with increasing excitation power density. The thickness of the InAs layers were estimated to be ~ 3 MLs for bands C and D, according to Fig. 2f. The exciton of the PL emissions at bands C and D, therefore, originated from free exciton emission in the 3ML-thick InAs layer, in which the photo-generated carriers are recombined via the ground state in InAs QW.

Figure 4c shows the temperature dependence of the PL spectra. The narrow PL emissions remained at the same intensity between 4.2 and 40 K, then decreased with an activation energy of ~ 52 meV for A1 and 61 meV for A2, and consequently quenched at ~ 70 K. The activation energy was estimated using an Arrehnius equation^35^. (The fitting results for bands A and D are shown in Supplementary Fig. S4.) The activation energy for band A corresponds to those of (111)-oriented InAs QDs^36^. The rate parameter for the A1 and A2 were 5.9 × 10^5^ and 1.75 × 10^6^, respectively. The large activation energy for band A indicates efficient electron–hole capture and radiative recombination in the 2D InAs island. The smaller rate parameter for band A1 means the thermal emission transport from the 2D into underneath the InAs layer was efficient.

The activation energy for other PL bands, such as B, was estimated to be 17 meV for bands B1 and 12 meV for B2 B2. In the band B, the activation energy for B1 was larger than band B2, both energies remained at 10 meV. This indicated that almost all photo-generated carries were thermally transport to underneath the InAs layers with increasing temperature. The activation energy for band D1 and D2 were estimated to be 13 and 32 meV, respectively, while the rate parameter was 50 and 380, respectively. These small rate parameters indicate that the radiative lifetime was shorter than those of PL band A, which was related with misfit dislocation of the GaAs/InAs heterointerface.

In conclusion, we have demonstrated selective-area regrowth of forming atomically-thin InAs layers in GaAs NWs integrated on Si platforms. The atomically thin InAs layers inside GaAs NW have exhibited sharp PL peaks originated from exciton emission of atomically thin 2D InAs islands. The selective-area regrowth involves specific early growth stage of 2D InAs islands with slow growth rate. By utilizing the extremely slow growth rate that reaches the nucleation process in the selective-area regrowth, the growth technologies would open new scheme in integrating the quantum regimes with Si platforms as building-blocks for versatile quantum systems.

## Methods

### Selective-area growth of vertical GaAs NWs

After the Si(111) was degreased with organic solvents, 20 nm-thick SiO_2_ film was formed by thermal oxidation. Hexagonal-shaped openings arranged in a triangular lattice with a pitch of 0.4–1 μm were then formed on the amorphous films by using electron-beam (EB) lithography and wet chemical etching. The opening diameter *d*_0_ was 70 nm. Metalorganic vapour phase epitaxy (MOVPE) was carried out to grow NWs. The GaAs NWs were grown in a horizontal low-pressure MOVPE system. Trimethylgallium (TMGa) and arsine (AsH_3_) gas were used as material sources. After thermal cleaning at 900 °C in H_2_, we carried out AsH_3_ treatment at 400 °C and low-temperature GaAs were grown for 3 min at 400 °C. Then, the GaAs NWs were grown for 60 min at 760 °C. The partial pressures of TMGa, AsH_3_ were respectively 1.0 × 10^–6^ and 2.5 × 10^–4^.

### Formation of GaAs/InAs layers for selective-area regrowth

Trimethylindium (TMIn) and AsH_3_ were used as the materials for InAs growth. The partial pressures of TMIn and AsH_3_ were 4.8 × 10^–7^ and 1.3 × 10^–4^ atm, respectively. The partial pressure of the GaAs was same as those of the GaAs NWs. The GaAs buffer layer was the same condition as the GaAs NWs. The InAs and GaAs were grown by pulsed growth. The growth temperature for GaAs/InAs multilayers were 560 °C. A cycle of the InAs and GaAs growth was carried out with Group III supply (TMIn or TMGa) for 1 s and AsH_3_ supply for 1 s with H_2_-intervals for 1 s.

### Microphotoluminescence measurement

Micro-PL (μ-PL) measurement was carried out at 4.2 K. The excitation light was He–Ne (632.8 nm) focused to a 2-μm spot using a 100 × objective lens. About ten NWs were inside the excitation laser spot at the same time. The PL spectra were taken with a nitrogen-cooled charge-coupled device detector.

## Supplementary information

Supplementary file1

## References

[1] Michler M, Kiraz A, Becher C, Schoenfeld WV, Petroff PM, Zhang L, Hu E, Imamoğlu A (2000). A quantum dots single-photon turnstile device. Science.

[2] Muller A, Fang W, Lawall J, Solomon GS (2009). Creating polarization-entangled photon pairs from a semiconductor quantum dots using the optical stark effect. Phys. Rev. Lett..

[3] Leonard D, Krishnamurthy A, Reaves CM, Denbaars SP, Petroff PM (1993). Direct formation of quantum-sized dots from uniform coherent islands of InGaAs on GaAs surfaces. Appl. Phys. Lett..

[4] Björk MT, Ohlson BJ, Sass T, Persson AI, Thelander C, Magnusson MH, Deppert K, Wallenberg LR, Samuelson L (2002). One-dimensional heterostructures in semiconductor nanowhiskers. Appl. Phys. Lett..

[5] Gudiksen MS, Lauhon LJ, Wang J, Smith DC, Lieber CM (2002). Growth of nanowire superlattice structures for nanoscale photonics and electronics. Nature.

[6] Zhang G, Takiguchi M, Tateno K, Tawara T, Notomi M (2019). Telecom-band lasing in single InP/InAs heterostructure nanowires at room temperature. Sci. Adv..

[7] Zhong Z, Li X, Wu J, Li C, Xie RB, Yuan X, Niu X, Wang W, Luo X, Zhang G, Wang ZM, Tan HH, Jagadish C (2019). Wavelegth-tunable InAsP quantum dots in InP nanowires. Appl. Phys. Lett..

[8] Leandro L, Gunnarsson CP, Reznik R, Jöns KD, Shtrom I, Khrebtov A, Kasama T, Zwiller V, Cirlin G, Akopian N (2018). Nanowire quantum dots tuned to atomic resonances. Nano Lett..

[9] Björk MT, Thelander C, Hansen AE, Jensen LE, Larsson MW, Wallenberg LR, Samuelson L (2004). Few-electron quantum dots in nanowires. Nano Lett..

[10] Li Y, Qian F, Xiang J, Lieber CM (2006). Nanowire electronic and optoelectronic devices. Mat. Today.

[11] Dheeraj DL, Patriarche G, Zhou H, Hoang TB, Moses AF, Grønsberg S, van Helvoort ATJ, Fimland B-O, Weman H (2008). Growth and characterization of wurtzite GaAs nanowires with defect-free zinc blende GaAsSb inserts. Nano Lett..

[12] Sun X, Wang P, Sheng B, Wang T, Chen Z, Gao K, Li M, Zhang L, Ge W, Arakawa Y, Shen B, Holmes M, Wang X (2019). Single-photon emission from a further confined InGaN/GaN quantum disc via reverse-reaction growth. Quantum Eng..

[13] van Weert MHM, Akopian N, Perinetti U, van Kouwen MP, Algra RE, Verheijen MA, Bakkers EPAM, Kouwenhoven LP, Zwiller V (2009). Selective excitation and detection of spin states in a single nanowire quantum dot. Nano Lett..

[14] van Weert MHM, den Heijer M, van Kouwen MP, Algra RE, Bakkers EPAM, Kouwenhoven LP, Zwiller V (2010). Surround-gated vertical nanowire quantum dots. Appl. Phys. Lett..

[15] Tanabe K, Watanebe K, Arakawa Y (2012). III–V/Si hybrid photonic devices by direct fusion bonding. Sci. Rep..

[16] Tanabe K, Watanebe K, Arakawa Y (2012). 1.3 μm InAs/GaAs quantum dot laser on Si rib structures with current injection across direct-bonded GaAs/Si heterointerfaces. Opt. Exp..

[17] Tomioka K, Kobayashi Y, Motohisa J, Hara S, Fukui T (2009). Selective-area growth of vertically aligned GaAs and GaAs/AlGaAs core-shell nanowires on Si(111) substrate. Nanotechnology.

[18] Tomioka K, Motohisa J, Hara S, Hiruma K, Fukui T (2010). GaAs/AlGaAs core multishell nanowire-based light-emitting diodes on Si. Nano Lett..

[19] Borg M, Schmid H, Moselund KE, Signorello G, Gignac L, Bruley J, Breslin C, Kanungo PD, Werner P, Riel H (2014). Vertical III–V nanowire devices integration on Si(100). Nano Lett..

[20] Knoedler M, Bologna N, Schmid H, Borg M, Moselund KE, Wirths S, Rossell MD, Riel H (2017). Observation of twin-free GaAs nanowire growth using template-assisted selective epitaxy. Cryst. Growth Des..

[21] Schmid H, Borg M, Moselund KE, Gignac L, Breslin CM, Bruley J, Cutaia D, Riel H (2015). Template-assisted selective epitaxy of III–V nanoscale devices for co-planar heterogeneous integration with Si. Appl. Phys. Lett..

[22] Wirth S, Mayer BF, Schmid H, Sousa M, Gooth J, Riel H, Moselund KE (2018). Room-temperature lasing from monolithically integrated GaAs microdisks on silicon. ACS Nano.

[23] Nishida T, Kobayashi N (1997). Formation of a step-free InAs quantum well selectively grown on a GaAs (111)B substrate. J. Electron. Mater..

[24] Bhattacharya P (1993). Properties of Lattice-Matched and Strained Indium Gallium Arsenide.

[25] Yamaguchi H, Belk JG, Zhang XM, Sudijono JL, Fahy MR, Jones TS, Pashley DW, Joyce BA (1997). Atomic-scale imaging of strain relaxation via misfit dislocations in highly mismatched semiconductor heteroepitaxy: InAs/GaAs(111)A. Phys. Rev. B.

[26] Kanisawa K, Butcher MJ, Yamaguchi H, Hirayama Y (2001). Imaging of friedel oscillation patterns of two-dimensionally accumulated electrons at epitaxially grown InAs(111)A surfaces. Phys. Rev. Lett..

[27] Ohtake A, Nakamura J, Komura T, Hanada T, Yao T, Kuramochi H, Oseki M (2001). Surface structures of GaAs{111}A, B – (2×2). Phys. Rev. B.

[28] Borg M, Gignac L, Bruley J, Malmgren A, Sant S, Convertino C, Rossell MD, Sousa M, Breslin C, Riel H, Moselund KE, Schmid H (2019). Facet-selective group-III incorporation in InGaAs template assisted selective epitaxy. Nanotechnology.

[29] Johansson J, Karlsson LS, Dick KA, Bolinsson J, Wacaser BA, Deppert K, Samuelson L (2009). Effects of supersaturation on the crystal structure of gold seeded III–V nanowires. Cryst. Growth Des..

[30] Jacobsson D, Panciera F, Tersoff J, Reuter MC, Lehman S, Hofmann S, Dick KA, Ross FM (2016). Interface dynamics and crystal phase switching in GaAs nanowires. Nature.

[31] Sköld N, Pistol M-E, Dick KA, Pyyor C, Wagner JB, Karlsson LS, Samuelson L (2009). Microphotoluminescence studies of tunable wurtzite InAs_0.85_P_0.15_ quantum dots embedded in wurtzite InP nanowires. Phys. Rev. B.

[32] Uccelli E, Arbiol J, Morante JR, Morral AF (2010). InAs quantum dot arrays decorating the facets of GaAs nanowires. ACS Nano.

[33] Rodt S, Heitz R, Schliwa A, Sellin RL, Guffarth F, Bimberg D (2003). Repulsive exciton–exciton interaction in quantum dots. Phys. Rev. B.

[34] Stier O, Heitz R, Schliwa A, Bimberg D (2002). Shape and composition effects on excitons and biexcitons in quantum dots. Phys. Stat. Sol. A.

[35] Fang Y, Wang L, Sun Q, Lu T, Deng Z, Ma Z, Jiang Y, Jia H, Wang W, Zhou J, Chen H (2015). Investigation of temperature-dependence photoluminescence in multi-quantum wells. Sci. Rep.

[36] Schuck CF, Roy SK, Garrett T, Yuan Q, Wang Y, Cabrera CI, Grossklaus KA, Vandervelde TE, Liang B, Simmonds PJ (2019). Anomalous Stranski–Krastanov growth of (111)-oriented quantum dots with tunable wetting layer thickness. Sci. Rep..

