# Supplementary material for: Rational synthesis of atomically thin quantum structures in nanowires based on nucleation processes
Source: Sci Rep. 2020 Jul 1;10:10720. doi: 10.1038/s41598-020-67625-y (PMC7329807; doi:10.1038/s41598-020-67625-y)
Supplement: Supplementary file 1 — Supplementary file1 [file 41598_2020_67625_MOESM1_ESM.doc]

**Supporting material for manuscript entitled “**Rational synthesis of atomically thin quantum structures in nanowires based on nucleation processes

Katsuhiro Tomioka, ^*,†,‡^ Junichi Motohisa,^†^ and Takashi Fukui^†^

**S1. PL measurements for arbitral position.**

**
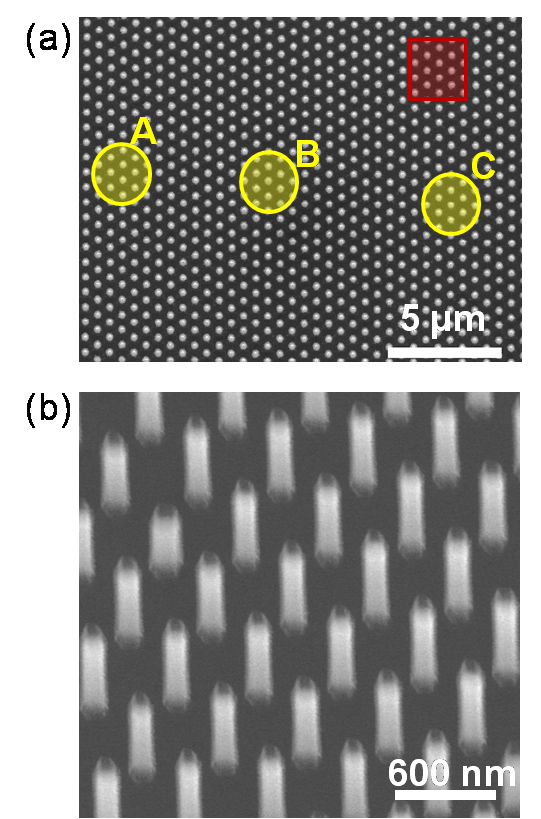
**

**Figure S1** (a) SEM image of GaAs/InAs/GaAs NW array on Si fabricated by regrowth method. The yellow circles show a position of excitation laser spot. About 10 NWs were inside the spot. (b) Magnified SEM image of GaAs/InAs/GaAs NW array as shown in red rectangle in (a).


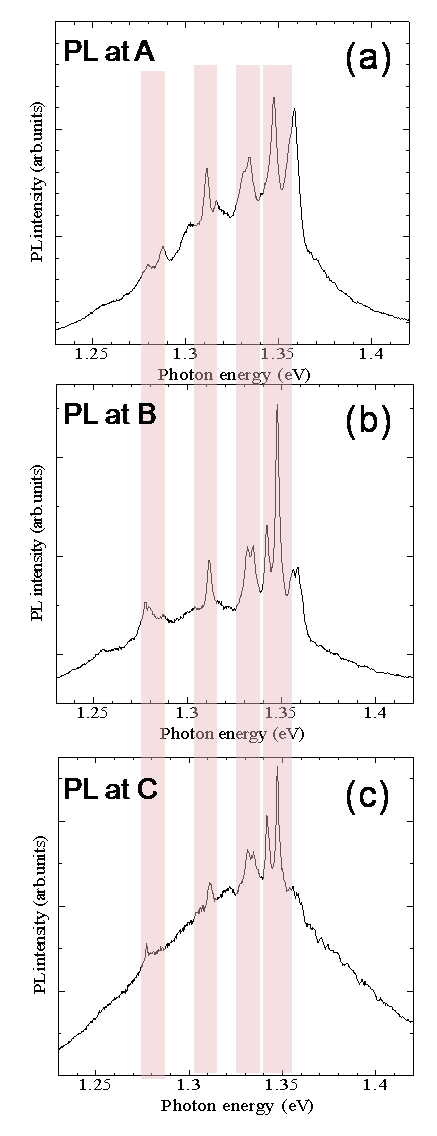


**Figure S2** PL spectra taken at position A, B, and C showed in Fig. S1(a). Red shaded band shows the PL peaks denoted as A, B, C, and D band in the manuscript. The peaks position of the PL bands didn’t changed and this shows size fluctuation of each GaAs/InAs/GaAs NW was significantly small.

**S2. Benchmark for recent related papers regarding single InAs QD on nanomaterials and planar surface**.

We listed representative papers regarding a single InAs QD on patterned nanomaterials such as nanowires and pillars and their PL properties in order to compare our structure with previous respectable reports as shown in Table S1. In this Table S1, we categorized the InAs/GaAs QD as growth/materials, host material, QD size (height/width), PL peak energy, FWHM of the PL emission, temperature, reproducibility, and position control. In Table S1, the InAs quantum structure by using our regrowth method showed similar emission phenomenon to the other reports, and had advantage in reproducibility and position controlling. These advantages guaranteed main argument of our method of forming InAs thin QD in position-controlled NW by nucleation process.

Table S1. Benchmark for recent related papers regarding single InAs QD on nanomaterials and planar surface


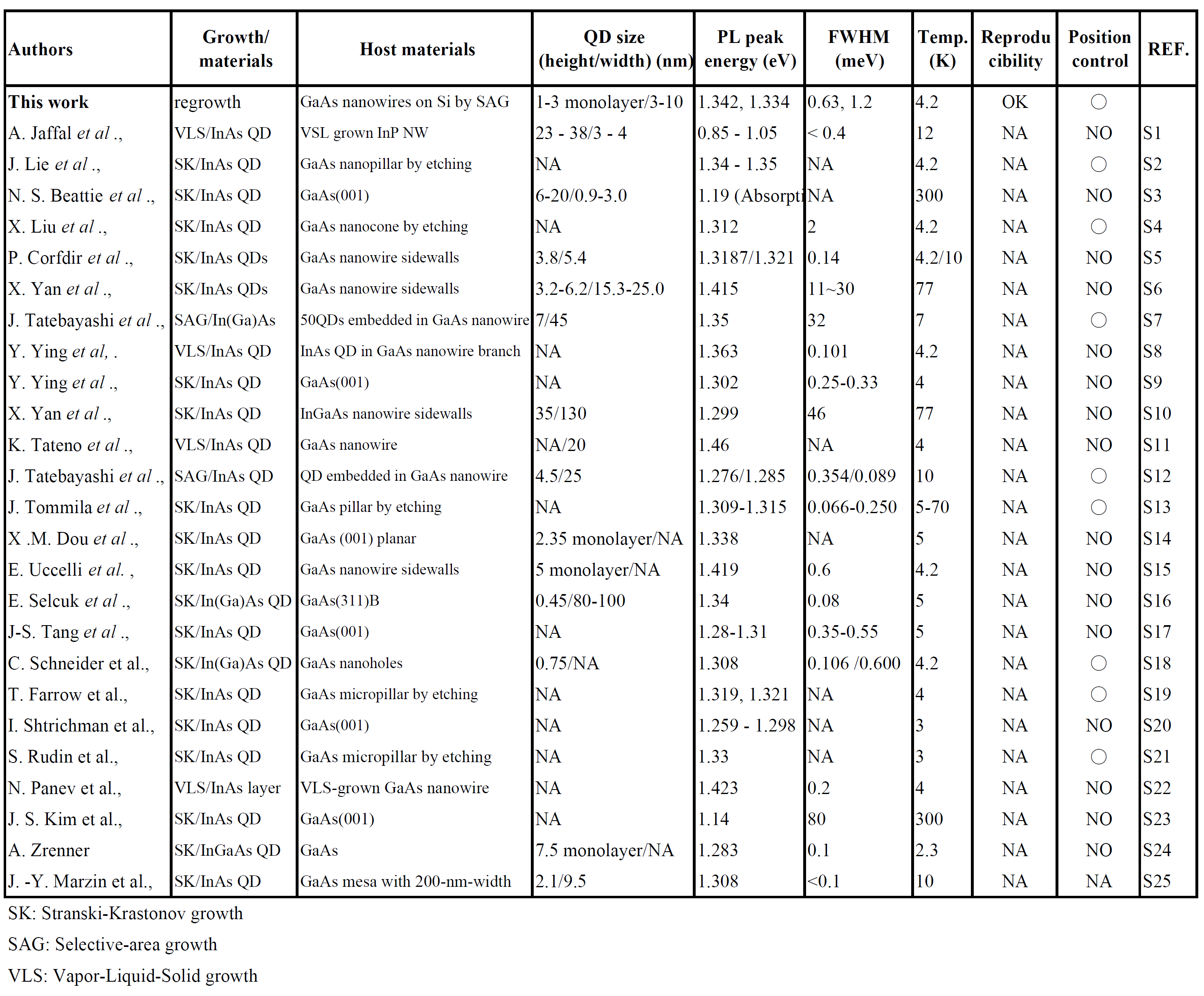


**References for Table. S1**

1. Jaffal *et al*., Nanoscale, **11** (2019) 21847 - 21855
2. J. Lie *et al*., Phys. Rev. Appl., **9** (2018)064109 -1 - 17
3. N. S. Beattie *et al*., ACS Photonics **4** (2017) 2745 - 2750
4. X. Liu *et al*., Opt. Exp. **25** (2017) 8073 - 8084
5. P. Corfdir *et al*., Phys. Rev. B **96** (2017) 045435-1 - 7
6. X. Yan *et al*., J. Appl. Phys. **117** (2015) 054301-1 - 6
7. J. Tatebayashi *et al*., Nature Photonics **9** (2015) 501 - 505
8. Y. Ying *et al*,. Nano Lett. **13** (2013) 1399 - 1404
9. Y. Ying *et al*., Appl. Phys. Lett. **102** (2013) 201103-1 - 4
10. X. Yan *et al*., J. Mat. Chem C **1** (2013) 7914 - 7919
11. K. Tateno *et al*., J. Nanotech **2012** (2012) 890607-1 - 8
12. J. Tatebayashi *et al*., Appl. Phys. Lett. **100** (2012) 263101-1 - 4
13. J. Tommila *et al*., Nanoscale Res. Lett. **7** (2012) 313
14. X .M. Dou *et al*., EPL **98** (2012) 17007-p1 - p5
15. E. Uccelli *et al*., ACS Nano **4** (2010) 5985 - 5993
16. E. Selcuk *et al*., Appl. Phys. Lett., **94** (2009) 263108-1 - 3
17. J-S. Tang *et al*., Physica W **41** (2009) 797 - 800
18. Schneider *et al*., Nanotechnology **20** (2009) 434012-1 - 9
19. T. Farrow *et al*., Nanotchnology **19** (2008) 345401-1 – 4
20. I. Shtrichman *et al*., Phys. Rev. B **65** (2007) 081303-1 - 4
21. S. Rudin *et al*., New J. Phys. **6** (2004) 89-1 - 16
22. N. Panev *et al*., Appl. Phys. Lett. **83** (2003) 2238 - 2240
23. J. S. Kim *et al*., J. Kor. Phys. Soc. **42** (2003) S476 - S479
24. A. Zrenner J. Chem. Phys. 112 (2000) 7790 - 7798.
25. J. -Y. Marzin *et al*., Phys. Rev. Lett. **73** (1994) 716 - 719

**S2. PL spectra for atomically thin InAs layers in GaAs NWs with cycles of 9, 18, and 30 cycles**.

The micro-PL spectra was measured from the GaAs NWs embedding in InAs layer grown by different pulse cycles. The InAs layers were grown with 9, 18, and 30 pulse cycles in order to characterize the nucleation and 2D InAs island layer from the TEM image. The typical PL spectra of the grown sample is shown in Figure S3. The diameter is 70 nm and length is approximately 700 nm. Except for the signal of the GaAs at 1.47 eV, the very narrow peaks at 1.322 eV, 1.326 eV, 1.302 eV and 1.240 eV can be seen in this PL under weak intensity excitation. The Full-Width-Half Maximum (FWHM) is 2, 3 meV. The PL peak at around 1.3 eV has also reported on InAs small island on GaAs [S26]. PL peaks at around 1.32 eV is resulted from the 3ML-thick InAs layer. If the very narrow PL signal assumes to be the luminescence of 2D InAs islands, the other peaks at around 1.30 eV and 1.24 eV presume to be those of thicker-InAs layers.

S26. S. Guha, A. Madhukar, and K. C. Rajkumar, Appl. Phys. Lett. **57**, 2110 (1990).

**
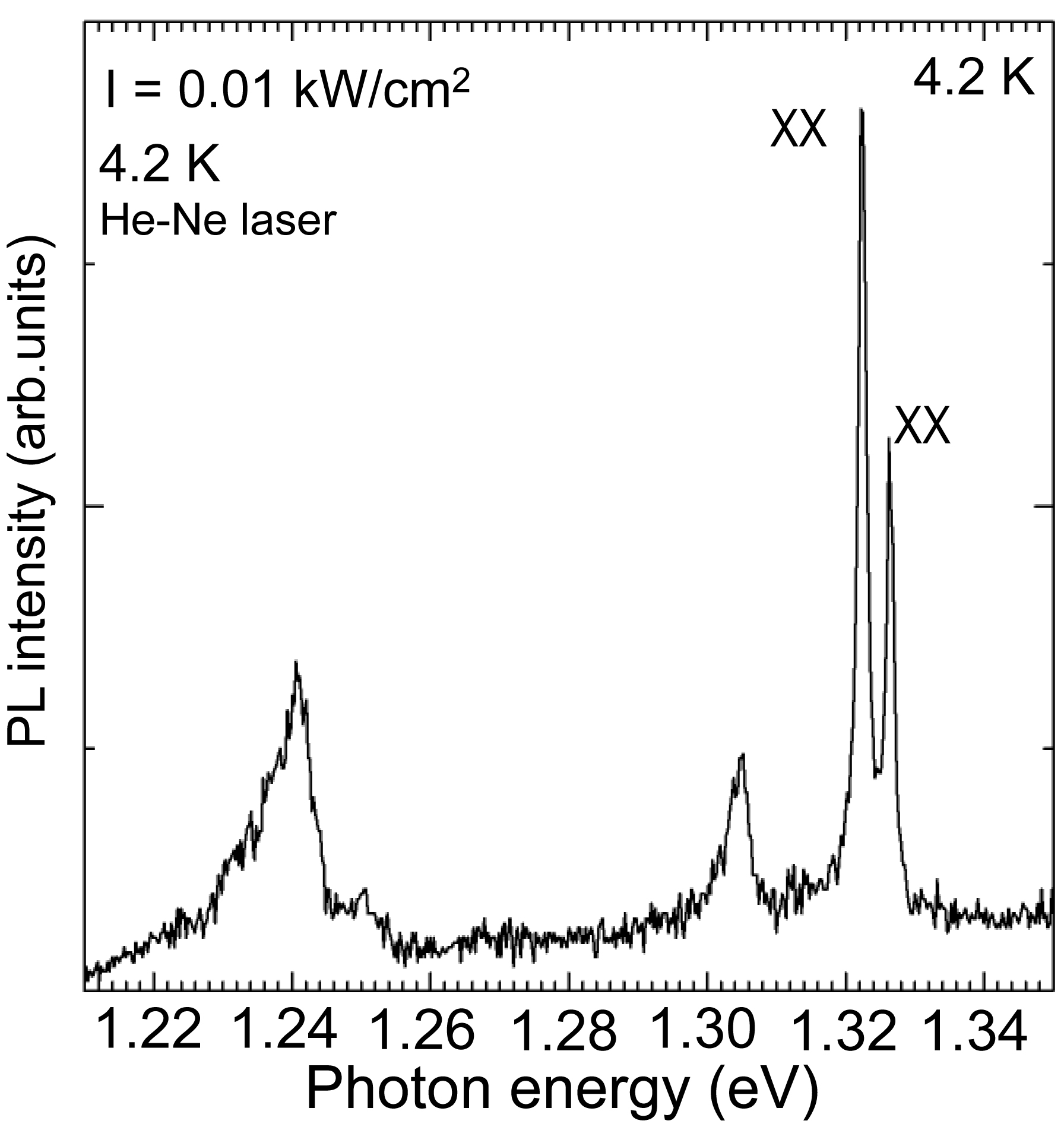
**

**Figure S3** PL spectra of atomically thin InAs layers embedded GaAs NWs, which was analyzed in Figure 3. The InAs growth cycles were 9, 18, 30 cycles.

]

**S3. Estimation of activation energy and rate paremeter from Arrehnius eauation.**

The activation energy was estimated using an Arrehnius equation^35^.

*I*(*T*) = *I*_0_/[1+*α* exp(-*E_a_*/*k*_B_*T*)] (1)

where *I*(*T*), *I*_0_, *α*, *E_a_*, and *k*_B_ are the integrated PL intensity at *T* K, a scaling factor, rate parameter, activation energy, and Boltzman constant, respectively


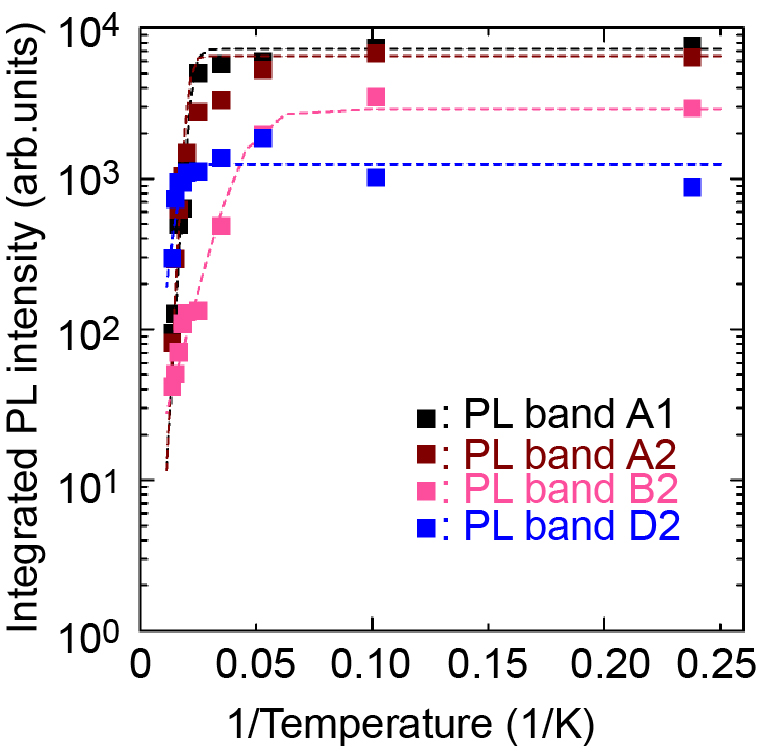


**Figure S4** Representative curve fitting for the temperature dependence of integrated PL intensities.

The dashed curves represent the best fit for the PL intensities as a function of 1/T, using a modified Arrhenius equation described in main manuscript.
